# Spontaneous rupture of renal angiomyolipoma presenting with shock; a case report from Tanzania

**DOI:** 10.1016/j.ijscr.2023.109073

**Published:** 2023-11-23

**Authors:** Nishi Chhatbar, Allyzain Ismail, Sajida Panjwani, Adil Datoo, Hussam Uddin, Aliakbar Zehri

**Affiliations:** a The Aga Khan Hospital, Dar-es-Salaam, Tanzania; b The Aga Khan University, East Africa Medical College, Tanzania; c Department of Radiology, The Aga Khan Hospital, Dar-es-Salaam, Tanzania; d Department of Urology, The Aga Khan Hospital, Dar-es-Salaam, Tanzania

**Keywords:** Angiomyolipoma, Spontaneous rupture, Shock, Case report

## Abstract

**Introduction and importance:**

Renal angiomyolipoma (AML) are benign tumors, often incidentally diagnosed with rupture being the commonest complication and cause of mortality. These tumors are rare with a higher prevalence among patients with tuberous sclerosis and female predominance. Management is dependent on tumor size and whether or not the tumor has ruptured.

**Case presentation:**

32-year-old female presenting with sudden right flank pain with shock without history of prior trauma or surgeries. Underwent imaging revealing a suspected ruptured AML thus underwent emergent nephrectomy with admission to intensive care.

**Clinical discussion:**

Wunderlich syndrome manifests as the Lenk triad, which includes acute flank pain, flank mass, and hypovolemic shock with signs of internal bleeding such as hematuria. It is a rare manifestation signifying spontaneous renal hemorrhage. Due to the instability of the patient had to undergo emergency laparotomy and nephrectomy.

**Conclusion:**

Renal AML occur as a rare benign phenomenon which when ruptured are associated with high mortality rate if not treated promptly in a setting with specialized treatment and intensive unit care. We hope that through our experience patients presenting with Lenk's triad are identified early for adequate intervention.

## Highlights

• Suspicion index for patients with Lenk triad
• Rarity of spontaneous rupture of angiomyolipoma
• Clinical implications for spontaneous tumor rupture
• Awareness of flank pain with hypotension

## 1 Introduction

Angiomyolipoma (AML) is a neoplasm with perivascular epithelioid differentiation composed of fat, smooth muscle and blood vessels. They generally present among 0.3 % of the population and more commonly affects females in their 5th decade of life [1]. They are either sporadic or associated with tuberous sclerosis complex (TSC) or lymphangioleiomyomatosis (LAM) [2]. Often, they are diagnosed incidentally but can be symptomatic when they rupture with a catastrophic presentations and outcome [3].

The diagnostic radiological suspicion for AML by is by non-contrast Computed Tomography (CT) which shows the presence of fat in a renal mass, defined in Hounsfield units (HU) as −10 (−15 to −30 HU). They can also be classified as fat rich, fat poor and fat invisible. This finding can also be seen in other renal masses such as renal cell carcinoma, lipoma, liposarcoma, oncocytoma, Wilms tumor, and teratomas. Therefore, other characteristics such as the contrast enhancement pattern and the presence of calcifications should also be considered [4]. Ultrasound has also shown some potential in identifying AML however due to user dependance variability and difficulty to differentiate from other renal masses, CT is the modality of choice [5].

An AML with a diameter of more than 4 cm is more likely to develop aneurysms thus making it more susceptible for spontaneous rupture and life-threatening bleeding [6]. However, a series of clinical studies have reported AML tumors less than 4 cm may also rupture spontaneously. We report on a case of a patient presenting with acute flank pain and hypotension in hemorrhagic shock due to spontaneous AML rupture. We aim to increase awareness on the lethal Lenks triad which can be due to a ruptured AML hence increase level of clinical suspicion needed in such patients with timely lifesaving intervention needed. This paper has been reported in line with the SCARE 2020 criteria [7]. This article has been registered with the Research Registry.

## 2 Case presentation

A 32-year-old female presented with sudden right flank pain for 2 h to the Accidents & Emergency Department without prior history of trauma. Also had nausea and vomiting however no episodes of visible hematuria, fevers, unintentional weight loss, easy fatiguability nor shortness of breath reported. She did not have any significant drug allergies, family history of malignancies, comorbid nor prior history of surgeries. She was nulliparous, on her third day of menstrual cycle, did not smoke nor consume alcohol.

On examination she was alert, afebrile, pale, not cyanotic, tachycardic with pulse rate ranging between 100 and 115 beats per minute and a blood pressure on the lower limit at 95/63 which improved to 105/69 with initial fluid bolus. Per abdominal examination revealed a normal contour, without surgical nor therapeutic scars. She had significant tenderness noted at the right flank without discoloration of overlying skin and positive right renal angle tenderness. Rest of abdomen was soft without palpable organomegaly, tympanic percussion note and normal bowel sounds.

**Fig. 1:**
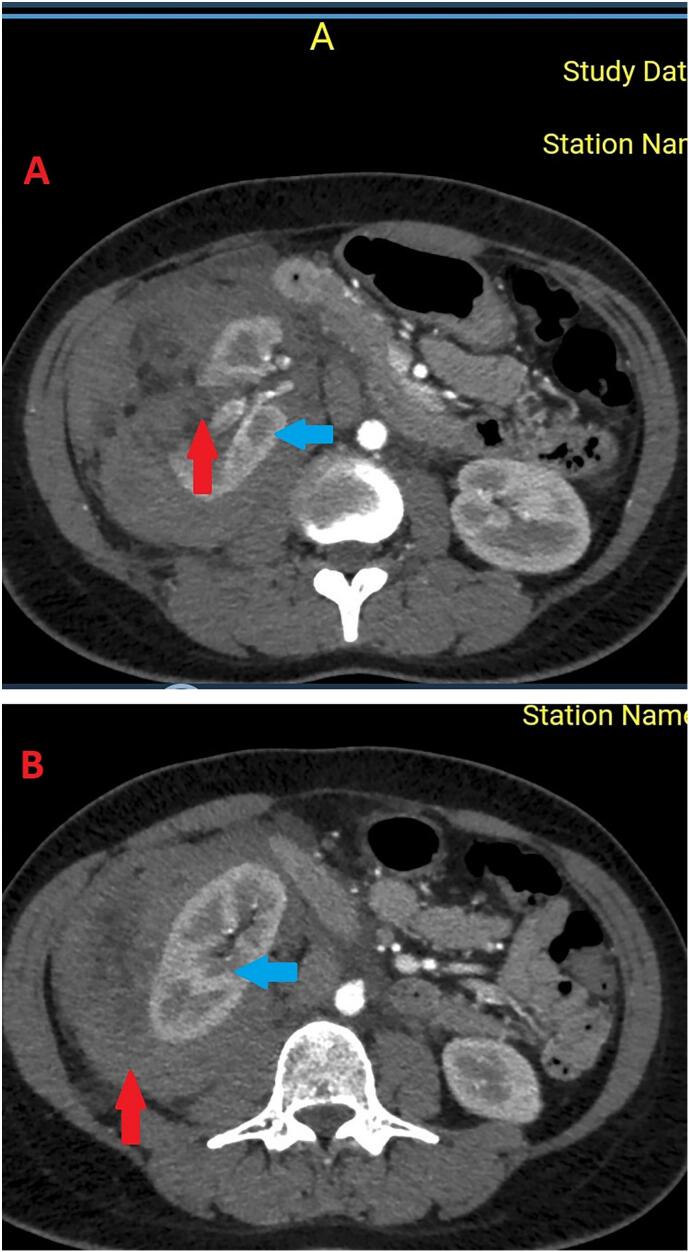
CT scan of abdomen with intravenous contrast axial view. A – Showing an exophytic lesion with a sentinel clot sign (red arrow) arrising from the right kidney (blue arrow). B – Showing a hematoma (red arrow) surrounding the right kidney (blue arrow). (For interpretation of the references to colour in this figure legend, the reader is referred to the web version of this article.)

Initial blood work up revealed leukocytosis predominantly neutrophilia with a hemoglobin level of 7.4 g/dL, elevated creatinine level of 102.13 umol/L, negative serum Beta HCG test, and urine analysis significant for 10–25 red blood cells/hpf. A contrasted Computed Tomography scan (Fig. 1) of the abdomen revealed a sentinel clot sign in proximity to a fat-based lesion of the right kidney and with a perinephric hematoma suggestive of retroperitoneal hematoma secondary to a ruptured renal mass, most likely AML. She was persistently tachycardic and blood pressures dropped after initial response to fluid challenges to 85/60 hence prepared for emergency laparotomy with preparations to mobilise blood products to transfuse.

**Fig. 2:**
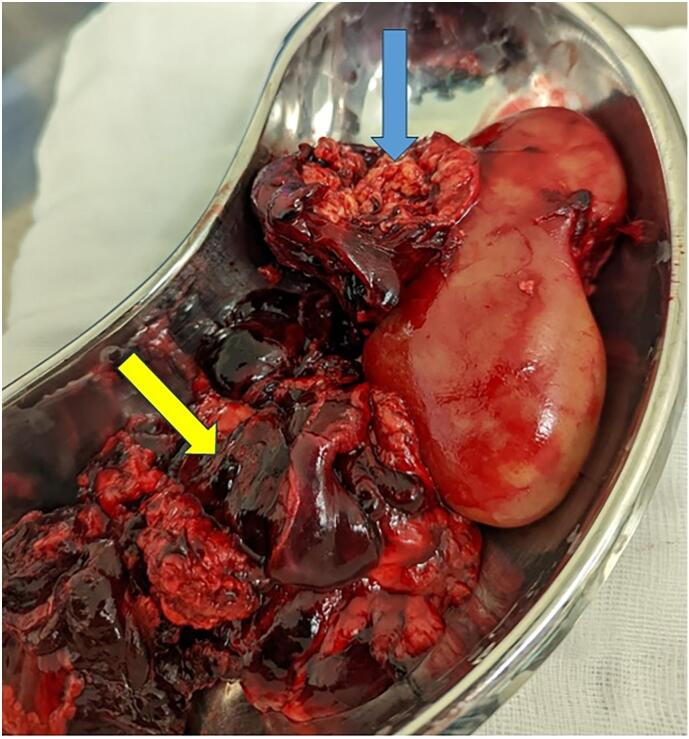
Right nephrectomy with an exphytic ruptured mass (blue arrow) seen with evacuated blood clots (yellow arrow). (For interpretation of the references to colour in this figure legend, the reader is referred to the web version of this article.)

A midline incision made with dissection into the abdominal cavity and duodenum kocherized to expose retroperitoneal contents. A retroperitoneal hematoma extending into the right paracolic gutter displacing overlying bowel to the left was seen which encased the right renal hilum as well as the inferior vena cava. Encased within the hematoma revealed a right kidney with fungating mass extending from the mid to the upper pole, suspicious of a ruptured AML (Fig. 2). The hematoma was opened and drained followed by clamping at the hilum of the right kidney for vascular control and right nephrectomy performed and sent for histopathology.

Post-operative was sent to the intensive care unit for close observation to receive 3 more units of blood, having received 2 units with 6 units of fresh frozen plasma at the emergency department, and fared well with no complications and vitals had normalised. Was transferred out by day 3 and by day 5 discharged home while tolerating regular feeds, ambulating and pain controlled with oral analgesia and a hemoglobin level of 11 g/dL. Follow up in the outpatient department was uneventful.

Histopathology showed benign renal parenchyma with tumor composed of mature adipose tissue, thick-walled dilated blood vessels which are congested and spindle (rhabdoid like) to round to oval cells with some pleomorphism, others with vesicular chromatin and prominent nucleoli. Few scattered mitoses were seen. Hemorrhage and necrosis were present with free ureter and renal hilar vessels. Hence confirming diagnosis of right ruptured renal AML.

## 3 Discussion

Wunderlich in 1856 first described the clinical picture of spontaneous renal bleeding confined to subcapsular or perinephric space in patients with no known underlying cause [8]. Acute bleeding, also called the Wunderlich syndrome manifests as the Lenk triad, which includes acute flank pain, flank mass, and hypovolemic shock with signs of internal bleeding such as hematuria [6]. As in our case she presented with sudden onset right flank pain with hypovolemia and despite not having overt gross hematuria had blood on urine microscopy hence the importance of suspecting bleeding in patients presenting with the Lenk triad.

Treatment of AMLs includes active surveillance, surgery (nephrectomy or partial nephrectomy), selective angioembolization, thermal ablation, or systemic therapy with mTOR (mammalian target of rapamycin) inhibitors [2]. Once a patient was diagnosed with spontaneous perirenal hemorrhage due to AML, thus the proper treatment choice depends on the patient's clinical status, laboratory findings, and degree of kidney rupture and the size of the retroperitoneal mass [9]. Due to our patient presenting with shock not responding to fluids, the decision to go for an explorative laparotomy and nephrectomy was carried out.

Upon completion of diagnostic evaluation, if indications for surgical resection are not present, active surveillance should be used to monitor progression in size and to identify development of new ones [10]. Nephrectomy is indicated only when a renal AML is very large, with high suspicion of malignancy, when other treatment options cannot be performed and when patient is unstable in shock. Ideally surgical resection should rely on a nephron sparing approach whenever possible. Parenchymal preservation is even more important in patients with TSC or LAM, because of the multifocal disease pattern and the higher recurrence rate in both kidneys. In an emergency setting however, nephrectomy can be lifesaving, as was the case with our patient [10]. For patients in hemorrhagic shock who are too unstable to be transferred to the interventional radiology suite, resuscitative endovascular balloon occlusion of the aorta is another life-saving bridge to embolisation therapy or emergent nephrectomy [11].

Selective Arterial Embolization (SAE) of renal artery is a safe and effective treatment for symptomatic and large AML. Most of AML treated with SAE show a mean reduction in size of about 43 %. However, in a minority of cases lesions do not shrink, instead they increase in size thus advised to repeated angiography and retreat the lesion by SAE [12]. However, its disadvantages include post embolization syndrome (fever and flank pain), need for re-embolization due to neo angiogenesis or recanalization of treated vessels, contrast material allergy and renal function impairment [13]. Ethanol denaturates proteins resulting in intravascular thrombosis and subsequent tissue infarction and is therefore widely used in embolization therapy [14].

Management of patients with spontaneous rupture is more individualized rather than the stepwise approach for an incidentally discovered AML. Factors based on hemodynamic stability and expertise availability dictates management hence due to the unstable nature of our patient we went in for an emergency laparotomy and nephrectomy so as to control the hemorrhage and facilitate adequate resuscitation with blood products in the ICU.

## 4 Conclusion

Renal AML occur as a rare benign phenomenon which when ruptured are associated with high mortality rate if not treated promptly in a setting with specialized treatment and intensive unit care. In our setting, there is under-reporting as well as missed diagnoses of renal AML, making our case report essential. We hope that through our experience of successful treatment approach to spontaneous rupture of AML, practitioners, particularly in resource limited settings, will be able to suspect individuals presenting with Lenk's triad so as to diagnose them early and manage them accordingly.

## Abbreviations

AML: angiomyolipoma
CT: Computed Tomography
HU: Hounsfield units
LAM: lymphangioleiomyomatosis
SAE: selective arterial embolisation
TSC: tuberous sclerosis complex

## Patient's perspective

I was very worried when I went to the hospital, everything was going so fast and being told I need an emergency operation was very stressful. However, looking back, I am very grateful to the team in charge of my care and can still not believe how having the tumor on my kidney made me so vulnerable.

## Informed consent

Written informed consent was obtained from the patient for publication and any accompanying images. A copy of the written consent is available for review by the Editor-in-Chief of this journal on request.

## Provenance and peer review

Not commissioned, externally peer-reviewed.

## Ethical approval

Written informed consent was obtained from the patient for publication and any accompanying images. A copy of the written consent is available for review by the Editor-in-Chief of this journal on request.

Ethical approval is not required at our institution (Aga Khan University, Tanzania) for case reports if patient particulars are not disclosed.

## Funding

This research did not receive any specific grant from funding agencies in the public, commercial, or not-for-profit sectors.

## Author contribution

N.C: Study conception, production of initial manuscript, collection of data, proofreading

A.I: Revision of the manuscript, proofreading

S.P: Revision of the manuscript, proofreading

A.D: Revision of the manuscript, proofreading

H.U: Production of initial manuscript, collection of data.

A.Z: Study conception, production of initial manuscript, collection of data.

## Guarantor

Dr. Aliakbar Zehri.

## Research registration number

1. Name of the registry: RESEARCH REGISTRY
2. Unique identifying number or registration ID: researchregistry9589
3. Hyperlink to your specific registration (must be publicly accessible and will be checked): https://www.researchregistry.com/register-now#userresearchregistry/registerresearchdetails/6524157f5a974c0028f90483/

## Conflict of interest statement

No conflicts of interest.
